# Social customer relationship management: taking advantage of Web 2.0 and Big Data technologies

**DOI:** 10.1186/s40064-016-3128-y

**Published:** 2016-08-31

**Authors:** Sergio Orenga-Roglá, Ricardo Chalmeta

**Affiliations:** Research Group on Integration and Re-Engineering of Systems (IRIS). Departamento de Lenguajes y Sistemas Informáticos, Unversitat Jaume I, Av. de Vicent Sos Baynat s/n, Castellón, Spain

**Keywords:** Social customer relationship management, Customer engagement, Web 2.0 technologies, Big Data technologies

## Abstract

The emergence of Web 2.0 and Big Data technologies has allowed a new customer relationship strategy based on interactivity and collaboration called Social Customer Relationship Management (Social CRM) to be created. This enhances customer engagement and satisfaction. The implementation of Social CRM is a complex task that involves different organisational, human and technological aspects. However, there is a lack of methodologies to assist companies in these processes. This paper shows a novel methodology that helps companies to implement Social CRM, taking into account different aspects such as social customer strategy, the Social CRM performance measurement system, the Social CRM business processes, or the Social CRM computer system. The methodology was applied to one company in order to validate and refine it.

## Background

The view that companies have of a customer has evolved dramatically with increasing competition, market globalisation and technological advances. Prior to the twentieth century companies focused only on production. During the first half of the twentieth century, however, companies began competing to persuade customers to buy their products. Later, during the second half of the same century, companies realised that they did not have to sell customers what they manufactured, but had to make what was demanded in market sectors. At the beginning of the twenty-first century, a stage began where the prevailing business orientation focused on customer relationship management (CRM), where each customer was treated individually and uniquely, depending on their preferences (Bose 2002).

The essence of CRM is to change the strategy of organisations, to move from a product-focused strategy to a customer-focused one. The aim is to create value for customers, understand their needs and offer value-added services (King and Burgess 2008). This increases the value of the company and allows it to gain a strategic advantage over competitors, because customers are more satisfied and, therefore, it is easier to retain them (Nguyen et al. 2007).

CRM has been conceptualised from five different viewpoints: (1) Process, (2) Strategy, (3) Philosophy, (4) Capability, and/or (5) Technological tools (Zablah et al. 2004). Therefore, there is no agreed definition of CRM. Among the most representative definitions is that provided by Bose (2002) “At the core, CRM is an integration of technologies and business processes used to satisfy the needs of a customer during any given interaction. More specifically, CRM involves acquisition, analysis and use of knowledge about customers to sell more goods or services and to do it more efficiently”. In this context, the work of Michael Fayerman (2002) should also be mentioned. This author distinguishes the following three areas of CRM within the company: (a) Operational CRM, which deals with actual interactions with customers; (b) Analytical CRM, which analyses data about a company’s customers and presents them in such a way as allow better and quicker business decisions to be made; and (c) Collaborative CRM, which promotes inter-departmental teamwork and communication within a company for the purpose of improving overall customer experience.

CRM is not just technology, as a proper implementation of CRM requires an integrated and balanced approach to technology, process and people (Chen and Popovich 2003). However, it has been the evolution of information technology and communications which has allowed its implementation. CRM links the systems of *front office* (sales, marketing, and customer service) and *back office* (financial, logistics, warehousing, accounting, human resources, and so forth) through the integration of technological and functional components (Strauss and Frost 2002; Zamil 2011). CRM mainly relies on the use of structured data from a data warehouse, where data are extracted, transformed and loaded from operation systems such as ERP, SCM or operational CRM.

In this context, the emergence of Web 2.0 technologies has allowed the evolution of CRM, which is based on a strategy focused on customer transactions, to Social CRM, which is based on a strategy focused on creating engagement between the customer and the company (offering new points-of-contact between the two, not only with the salesperson, and building stronger customer links with the company) (Faase et al. 2011). Yet, Social CRM does not replace CRM, but complements it. Customer engagement using Web 2.0 technologies is only possible when there is already a customer management using CRM (Faase et al. 2011). What Social CRM adds are social features, functions, processes and different forms of interaction between the company and its customers, suppliers and partners (Greenberg 2010).

The social customer is the customer that makes use of social software, which moves in a scenario characterised by permanent connectivity, mobility, being multi-channel and the progress of the Internet of Things. The publication of opinions on the Internet allows customers to share their points of view about a product or service. Companies participate in the social network of users connecting to its target group. This facilitates the opportunity to gain business-relevant insights from the data accessible from the communication among users. These electronic word-of-mouth statements are very important for organisations, because it is a way (1) to know how customers perceive their products and/or services, (2) to intensify the relationship, and (3) to align the business with consumer needs (Rosenberger 2015). This scenario is a new model of interaction between people, which is being transferred virally to the relationship between customer and company. Unlike other technological revolutions, this change in the way they relate is not being led by the companies, but the customers and their expectation that the companies with which they relate operate a significant change in their access models and behave in accordance with this new social reality. This new model represents a business opportunity for companies in their customer management. As a workspace, it represents a challenge for companies, because it is necessary to manage both human information, which is characterised by being complex, unstructured, ubiquitous, multi-format and multi-channel, and also the traditional information.

Moreover, Social CRM benefits from Big Data because it facilitates more accurate decision-making and a more efficient distribution of knowledge among the social customers and the company (Anshari et al. 2015). Big Data technology can be used for many purposes in Social CRM. Some of these include: (1) *Commercial recommendation*, suggesting the product or service with greater probability of success for each customer; (2) *Competitive intelligence*, showing real-time automated information customised to the situation created by the customer, thereby allowing the company to maintain a contextualised dialogue and to obtain real-time information needed to make suitable decisions; (3) *Debt recovery of customers* from public information sources; (4) *Automated categorisation and routing of customer interactions* over any channel; (5) *Predictive models* of trend (purchase, abandonment, non-payment, etc.) and clustering of customers; and (6) 360° *customer view*, showing the relevant customer information performed through any channel and format.

The amount of open information available online from heterogeneous sources and domains is growing very quickly, and constitutes an important body of knowledge to support Social CRM. These data sources may disclose significant business opportunities and competitive advantage to those who are able to understand and leverage their value (Torre-Bastida et al. 2015). They can infer valuable information as a support for customer-related decision-making. Therefore, Big Data and Web 2.0 technologies could change Social CRM from an unavoidable tool to keep/gain the new segment of “social customers” into a business opportunity and a competitive advantage.

However, the development and implementation of Social CRM in a company is a complex task that involves different organisational, human and technological aspects (Bebensee et al. 2011; Rosenberger 2015). In order to assist in a process of this kind, a methodology for managing the innovation and change involved in Social CRM is needed, while also reducing both the risk of failure in the implementation, as well as the time required to obtain business benefits (Crockett and Reed 2003; Lech 2016; Nguyen et al. 2007).

The literature about Social CRM does not contain any specific methodologies to help in the development of Social CRM. The research that exists on the topic of Social CRM focuses primarily on the characteristics, opportunities and benefits that Social CRM offers (Faase et al. 2011; Greenberg 2010; Mosadegh and Behboudi 2011) but does not offer any methodological guidelines.

To help solve this problem, a methodology called SCRM-IRIS is presented in this paper, which guides the development and implementation of Social CRM in a company. The rest of the paper is organised as follows. Section two shows the literature review. In section three the research method that was followed to obtain the methodology is shown. Section four presents the methodology proposed for the implementation of Social CRM. Finally, section five presents the conclusions.

## Literature review

### Social software

There is no generally agreed definition of Web 2.0. One of the most widely used is that proposed by Tim O’Reilly, who defines it as “a set of economic, social, and technology trends that collectively form the basis for the next generation of the Internet—a more mature, distinctive medium characterised by user participation, openness, and network effects” (Musser et al. 2006, p. 4). Web 2.0 is not only a new generation of technologies, but also a change in the way in which users access the Internet in order to mutually interact and collectively create knowledge. Some of the most common Web 2.0 tools include: Wikis, Group chats, Social bookmarking, Mashups, Blogs, RSS, Folksonomy, Podcasts and Social Networks.

Social software (which consists of the applications created with Web 2.0 technologies for social purposes) enables the development of new communication tools that allow a competitive advantage to be created in organisations (Wirtz et al. 2013). In social media (which is the set of social software applications), users can find not only information, but are active contributors (Lai and To 2015; Razmerita et al. 2009) and can freely express their comments, views and emotions (Feng et al. 2011). Therefore, social media encourage the creation, sharing and exchange of data. As stated above, there is a large variety of types of social software applications, such as Social Networks (which allow social capital to be managed more efficiently), Blogs (to communicate with others more effectively), Wikis and Social bookmarking (to make better use of collective intelligence), Group chats, Mashups, Multimedia Sharing, RSS, Folksonomy or Podcasts. These technologies are open and are designed to encourage collaboration as well as to facilitate social interaction and the sharing of knowledge (Dietrich et al. 2008; Kirchner et al. 2009; Ras and Rech 2009).

Social software only provides the framework, the content is provided by people (Omerzel 2010). Furthermore, the number of people using social software is very important. As more people use these applications, the overall value of knowledge will be significantly increased, i.e. collective intelligence will increase (Shimazu and Koike 2007). Users provide data and services in a way that allows others to combine them again, thus creating a network of effects through the “architecture of participation” (O’Reilly 2005). Recently, the social media have become a strategic tool for organisations, since they allow companies to meet the needs of customers as well as to provide them with new services (Go and You 2016).

### Social CRM

CRM can take advantage of social media, whose relational properties and characteristics are particularly well suited to customer interactions (Olbrich and Holsing 2012). Social CRM can be defined as “A philosophy and a business strategy, supported by a technology platform, business rules, processes and social characteristics, designed to engage the customer in a collaborative conversation in order to provide mutually beneficial value in a trusted and transparent business environment. It’s the company’s response to the customer’s ownership of the conversation” (Greenberg 2009). This definition includes the central principle of *customer engagement*, which was missing in earlier CRM models, and social media technologies facilitate this customer engagement (Olbrich and Holsing 2012).

Therefore, for a Social CRM system to work, there must be an important cultural and behavioural change both in the company as well as in the customers, as they have to change the way in which they interact (Greenberg 2009). Contribution, sharing, collaboration, dynamism and bidirectional trust between the company and customers become fundamental aspects in Social CRM (Lee and Lan 2007). The concept of social customer thus appears, which can be defined as a new type of customer that uses social software to search for, compare and exchange views on products and services offered by a company, and who expects companies to not only be present in that social software but also to respond to questions and participate. This customer acquires knowledge about new products and services through social channels and networks, prefers a conversation with the particular brand rather than it being just a way to send messages and at the same time wait for an answer, and wants the company to listen to and solve their problems quickly.

The social customer creates a new business model, called social business, which can help companies increase their profitability because it allows a number of qualitative and quantitative benefits to be obtained. The qualitative ones include: a better understanding and interpretation of the market, by interacting with customers in real time; benefiting from word of mouth; involving and engaging the customer at all stages of development of the product or service offered by the company (design, production, testing, etc.); improving the overall customer experience and lifetime value; enhancing products and services, or building up trust (Faase et al. 2011; Mosadegh and Behboudi 2011; Reinhold and Alt 2011; Sarner et al. 2012). Some the most significant quantitative benefits that could be achieved with the use of Social CRM are: increased sales; decreased service costs; reduced or replaced direct costs of printing and online advertising; reduced direct staff time costs; increased direct revenue from memberships, registers and advertising, exhibitions and sponsorship (Dreyer and Grant 2011b).

However, this new business model has a number of risks for companies (Assaad and Marx Gómez 2011). Both good and bad news spread quickly; social software is not well controlled or censored, so anyone can publish anything good or bad about the company or its products or services; and problems regarding personal privacy and security can emerge as the user is required to share at least some personal data.

### Big Data

Moreover, Social CRM benefits from Big Data, which is based on the current ability to have a large amount of data and draw conclusions about all sorts of company-customer processes and interactions. The digital world, mobility, and permanent connectivity have completely changed these processes and interactions over the last two decades. In addition, advances in infrastructure, storage techniques, and data-processing allow these huge volumes of structured and unstructured customer data to be analysed in a very fast and efficient way, and with an acceptable cost for most organisations. Due to the amount and complexity of these data, it is difficult to process them using traditional tools, so the use of Big Data technology is essential in order to take advantage of this kind of data (Syed et al. 2013).

Big Data technology is able to overcome the difficulties involved in understanding and extracting relevant knowledge from different kinds of data, which include: Diversity in types of fonts, formats and languages; Unstructured information (ideas, emotions, nuances, ambiguities, polysemy, etc.) that is contextual and has complex and fuzzy relations, such as distance, overlap, correlation, similarity, opposition, etc.; Dependence on the context in which it is emitted; Semantic problems due to the fact that language is constantly changing; and Dependence on grammar, language and the medium used.

A more subtle aspect of Big Data which is not frequently mentioned is that the analysis of massive data, which are often incomplete and even slightly inaccurate, seeks to find correlations and detect “things that are happening”, largely ignoring the analysis of causality. The emphasis is on the “what”, not the “why”. However, the growing analytical arsenal and existing advanced modelling techniques applied to massive datasets by professionals with appropriate levels of creativity and expertise are currently reaching an enormous degree of success in discovering correlations in previously unknown customer knowledge.

### Big Data in Social CRM

Big Data is a technology with a real ability to transform very significant aspects of customer relationship management, thereby providing companies with a competitive advantage over its competitors. Big Data technology allows knowledge to be extracted from customer information and converted, in an effective, secure and scalable way, into real business value. From customer information and through Big Data, a company is able to reveal hidden knowledge of the customer, turning it into opportunities to maximise the business value of each customer, to act preventively, to improve customer satisfaction, to identify new opportunities, or to predict their tendency and intention profile.

It is noteworthy that companies are harnessing the power of Big Data and analytics to apply it in customer relationship management (Marshall et al. 2015). The business value is derived from the knowledge generated, once it is transferred to the design of products or services, to the segmentation of customers and markets, to the acquisition of new customers, to the understanding of customers, to the evolution of the portfolio, to the optimisation of any of the internal procedures and production processes, or to the changing way companies relate with employees, citizens, suppliers, partners or customers.

## Research methodology

Since Social CRM complements CRM, in order to obtain a Social CRM methodology, called the SCRM-IRIS methodology, that guides the development and implementation of Social CRM in a company, an initial version was first developed based on the CRM implementation methodology presented by Chalmeta (2006). This methodology was supplemented, adapted and updated based on the review of the existing literature on Web 2.0, Big Data, CRM and Social CRM, as well as on the experience of the authors. This initial version was then applied to one company with the aim of analysing, validating and refining it. In order to carry out the application, a work plan based on the case study methodology proposed by Runeson and Höst (2009) was followed. This consists of the following stages: Design and planning of the case study; Preparation for data collection; Collecting evidence; Analysis of collected data; and Validation of collected data. Each of them is described below:

### Design and planning of the case study

The aims of the case study are: (a) to validate the SCRM-IRIS methodology by verifying and confirming its usefulness, accuracy and quality, and (b) to refine and improve the methodology developed initially from the experience acquired by the researchers, the feedback obtained from the company involved, and the conclusions drawn in the case study.

The research work was conducted over a period of 10 months. The first task was to select the company in which the case study was to be applied. The criteria underlying the selection of this company were essentially: (1) a willingness to collaborate in the research, and (2) the fact that the management of this company was considering the idea of improving the efficiency of their customer relationship management using Web 2.0 and Big Data technologies. The selected company was a SME from the metal sector with a workforce of 250 employees. Their target customer ranges from large supermarkets to little grocery stores and individuals, from all over the world. It is important to note that this company was already using a traditional CRM application.

### Preparation for data collection

To begin the research work, an introductory series of group interviews were held in the company. The presentation focused on the basic points of a Social CRM project and, at the same time, the methodology that was going to be used (initial version of the SCRM-IRIS methodology) was also explained to them.

In order to undertake all the research tasks during the application of the methodology in the company, a mixed work team was set up with members that came from both the IRIS Research Group and the Social CRM team of the company. The company Social CRM project team was made up of five area managers, representing the main areas of the company: General management, Commercial management, Financial management, Technical management and Operations management.

### Collecting evidence

The data collected were the results of applying the different stages of the initial version of the SCRM-IRIS methodology to the company. Qualitative data were used, which were collected through direct methods (using an assortment of questionnaires and templates) and independent methods (copies of the documents and reports used in the company).

The questionnaires were answered by IRIS researchers during individual interviews with Social CRM project team members. Once the implementation of each of the nine phases that compose the SCRM-IRIS methodology had finished, the IRIS researchers interviewed the five area managers from the Social CRM project team on an individual basis. These interviews lasted approximately 20 min and were open (thus allowing interviewees to give a wide range of answers) and semi-structured (the questions were planned only as a guide, not to be asked in that same order, thereby allowing both the interviewers and the interviewees to improvise). The aims of these interviews were: to analyse the execution of the phase, to obtain feedback about the experience and the observations of each manager in each phase, to detect errors, and to collect proposals for improvement to the SCRM-IRIS methodology from each of them. There was a different questionnaire for each phase, and those questionnaires were common to all interviewees. Table 1 presents an example of the questionnaire followed by the IRIS researchers to conduct the interviews after the process map phase.

**Table 1:** Interview questionnaire for the process map phase

Interview questionnaire	
Phase: process map	
1	What new business processes have been created in your department at this phase?
2	What existing business processes have been improved in your department at this phase?
3	Are there any business processes that have not been considered at this phase?
4	What information does Social CRM offer that you did not have before?
5	How do you think the company benefits from new or modified business processes?
6	Has the company assigned the necessary resources for a successful implementation of this phase?
7	Has the researcher provided the necessary means for a successful implementation of this phase?
8	Have you missed the collaboration of someone or something in the implementation of this phase?
9	What problems have you noticed in the implementation of this phase?
10	What would you change or improve in the implementation of this phase? How would you do it?
11	Is there any information that has not been considered at this phase and that you think should have been taken into account?
12	Is it worth the effort invested in the implementation of this phase in view of the expected result?
13	Have the desired results been achieved in the estimated time?
14	What is your general opinion about the implementation of this phase?

Most comments obtained were positive, indicating that the SCRM-IRIS methodology guided them perfectly throughout the implementation of all the phases and made them consider things that had not been proposed so far, such as for example a strategic focus on social customer engagement, the social customer profile that they should lead, and they had to take into account the average age of users. They were also very surprised by the amount of information that could be obtained about social customers. Moreover, negative comments were taken into consideration to improve the methodology, such as the lack of a company social media policy and guidance on how the employees had to use social software, besides training them in legal issues, and the need for different levels of segmentation based on communities and sub-communities. In addition, some negative comments said that once Social CRM was implemented, all possible tasks related to its use should be carried out by low-level staff, as their labour costs were lower, but they must be properly trained and high-level staff must support them when they needed it.

Once the project had finished, meetings were held with the Social CRM project team in the company in order to enrich the initial SCRM-IRIS methodology by modifying/incorporating/removing phases, tasks, tools, and so on. After this process of revising the initial methodology, it was enhanced by incorporating all the contributions detected and then validated with the general agreement of the Social CRM project team.

### Analysis of data collected

The persons responsible for the application of the SCRM-IRIS methodology in the company indicated that the use of this methodology has allowed them to have an excellent view of the needs, scope, consequences and opportunities of the project, as well as allowing them to implement Social CRM quickly and without any significant problems. They also indicated that this has enabled them to have greater control over the project, because all the steps to be performed in each stage of the implementation are clearly defined.

On the other hand, the following benefits have also been highlighted by the Social CRM team of the company as being the most important provided by Social CRM:

- Centralisation of knowledge relating to the company’s customers in an accessible (for both internal users and users outside the company) and easy-to-use system, allowing a constant flow of that knowledge.
- Quick compilation and dissemination of information relating to customers.
- Allows an exchange of customer portfolios between salespeople that is quick, easy and reliable, because the system centralises all the knowledge about customers, including the historic features, preferences, movements, etc.
- It records all the history of queries and problems from customers with the solutions that were adopted. This history is available to answer queries or similar problems (for that client or others) more efficiently and requiring less time.
- Users can access the information they need at the time and place where they need it (even in real time). Furthermore, such information is always up to date.
- Decrease in the use of other communication channels (e.g. e-mail, phone, etc.), as the Social CRM enables more effective communication.
- Decrease in the work undertaken by the company’s employees due to: (1) the simplicity, speed, centralisation, efficiency and control provided by Social CRM, and (2) customers can manage different tasks on their own, for example, can track their orders, can make or change their orders, etc.
- It allows potential social customers to be found quickly, as well as the tracking of current social customers.
- It allows the company to know in real time what people think about the products and/or services offered by the company, or by their competitors.

After a year using Social CRM, a comparison of the value of some indicators with the value obtained a year before the implementation of Social CRM was performed. In this comparison, some significant increases can be observed due to the introduction of Social CRM in the company. The most significant increases are: New supermarket customers (3 %); New grocery store customers (18 %); New individual customers (27 %); Customer loyalty (11 %); Customer satisfaction (24 %); Amount of sales (13 %); Amount invoiced (10 %); and Presence in new countries (33 %).

### Validation of the data collected

As the data collected was qualitative, it was analysed using qualitative data methods of analysis. In this case, the analysis was inductive and was carried out parallel to the data collection, as it was performed after each of the stages that make up the SCRM-IRIS methodology had finished. The purpose of this was to be able to react quickly to the assessments encountered during the analysis of each stage and thus rectify each one of them and take advantage of these improvements in the following stages.

Any threats to the validity of the case study were reduced by using the Lincoln and Guba model (Robson 2002), in which five strategies are proposed for use in data collection to tackle three types of threats to validity. The three types of threats considered were *reactivity* (the researcher’s presence can affect the setup of the study), *researcher bias* (the researcher’s preconceived ideas can affect the way the researcher asks questions or interprets answers) and *respondent bias* (the researcher’s influence on the attitude of the people being studied) (Karlström and Runeson 2006).

With regard to the five possible strategies, in the present case study they were considered in the following way in order to make the results valid: (1) *Prolonged involvement*: the researcher is familiar with the environment being studied (in this case study, the researchers and the company had already been collaborating on previous projects). (2) *Triangulation*: the application of several methods in the study of a single object. In this case study, four types were considered: *Spatial triangulation of data* (three sources of data were considered: observation, interviews and documentation); *Personal triangulation of data* (all the members of the company Social CRM project team were interviewed in order to obtain information from each of them); *Investigator triangulation* (the interviews were conducted by a researcher and reviewed by another researcher); and *Theoretical triangulation* (the different points of view of the members of the Social CRM project team were taken into account). (3) *Member checking*: obtaining feedback from the people who are interviewed (in the case study, after each interview, a report containing the relevant information from the interview was checked by each interviewee). (4) *Negative case analysis*: attempting to find another explanation that differs from the one initially assumed for the observed phenomenon (here, the two researchers were working separately (investigator triangulation)). (5) *Audit trail*: keeping a record of all the documentation of the project so as to be available in the future.

## SCRM-IRIS methodology

The methodology for the implementation of Social CRM presented in this paper is based on the proposal by Chalmeta (2006) for CRM. This methodology, called CRM-IRIS, is organised in nine phases and helps during the process of developing and implementing a CRM System. It considers and integrates various aspects, such as defining a customer strategy, re-engineering customer-oriented business processes, human resources management, computer system, management of change or continuous improvement.

**Fig. 1:**
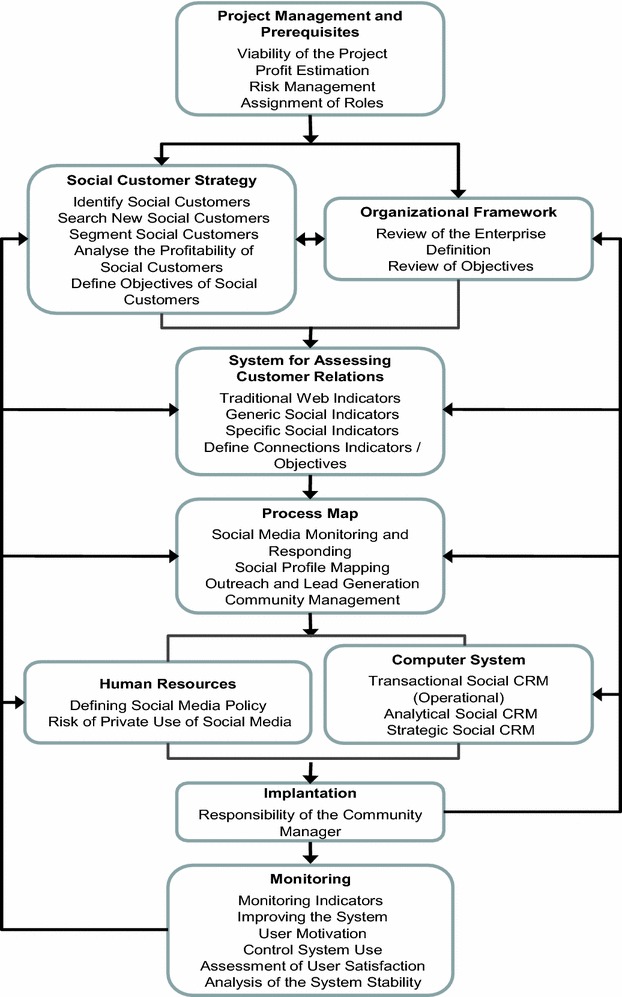
Additions and modifications to the CRM-IRIS methodology in order to consider Social CRM

The SCRM-IRIS methodology does not replace the CRM-IRIS methodology, but instead complements it, in order to adapt it to the features of Web 2.0 and Big Data technologies. It has not been necessary to add or remove phases, modify their sequence of application or delete the previous activities inside each phase. However, new activities inside each phase and modifications to some of the previous activities of the CRM-IRIS methodology have been added. Figure 1 shows these additions and modifications.

### Project management and prerequisites

There are no substantial differences in the project management between a Social CRM Project and a CRM Project, both of which must be managed as an engineering project. However, in the Social CRM Project the following basic prerequisites for success should be taken into account:

- *Viability of the project* Before starting the project there should be an analysis of whether Social CRM can be viable in the company, considering different aspects such as whether Social CRM is appropriate for the sector in which the company operates, the technological level of the company and its capacity to increase it, the ability of users to use social software applications, resistance to sharing knowledge and resistance to technological change especially in older employees and customers, etc. (Assaad and Marx Gómez 2011), as Social CRM will change the way of working (Dreyer and Grant 2011a).
- *Profit estimation* An estimate of both the quantitative and qualitative benefits expected to be achieved with the implementation of Social CRM must be carried out. When estimating benefits it is very important to think of not only the social objectives such as the number of fan pages and weekly tweets but to correlate Social CRM with the contribution of top business objectives. It is necessary to estimate return on investment (ROI), business value and budget justification for social projects before developing it.
- *Risk management* A risk assessment of the Social CRM must be carried out during this stage of the project in order to determine what issues need attention. First, the risks are identified, such as: possible misuse of the social software, posting of negative viral messages, privacy management, security of information, publication of private or confidential information, publication of misleading or false information, posting of negative comments, etc. Afterwards, every risk is assessed in relation to the frequency with which they may occur compared to the potential negative effect (financial, security, image, privacy, etc.) if they happen. Finally, priorities are established to mitigate the risks, addressing first the most severe and frequent events (Dreyer and Grant 2010). Once the risks have been understood, the methods to be used to manage them must be defined, such as for example (Dreyer and Grant 2010): Defining policies for users; Monitoring the social website to find out what is being said; Educating users on legal issues such as copyright and anti-trust; Educating users on the principles of social media; or Updating insurance policies to cover social media work.
- *Assignment of roles* The role of Community Manager must be assigned to an employee (or several employees) of the company (the marketing manager is recommended). His/her duty is to manage, build and moderate existing communities in the company by committing to social customers, making them feel part of the company and motivating them to take action, both on their own platforms, as well as using other public social software. Depending on the company’s structure, there may be several Community Managers located in different departments (usually in membership, communications or government relations) who collaborate with each other (Dreyer and Grant 2011a). It is also necessary to consider whether other roles need to be created (such as content creator, data analyst, and so on) to cover certain duties and to work with the Community Manager on social media projects (Dreyer and Grant 2011b).

### Organisational framework

In this phase, the analysis of the company’s objectives and culture should be performed taking into account the different characteristics of Social CRM:

- *Review of the enterprise definition* The aim, vision, strategy, policy and values of the company should be reformulated, taking into account how the company sees and manages its customers to enhance the benefit of both parties. The following key aspects of Social CRM should be taken into account: (1) Corporate strategy has to consider social customer (Greenberg 2010). (2) The relationship between business and the social customer focuses on a collaborative effort, and on social customer engagement and commitment, not social customer management (Greenberg 2010; Mosadegh and Behboudi 2011). (3) Customers are immune to the complexity of the departments of the company, so that all departments should manage customers. (4) The customer is the one who sets the schedule, because if the company is not responding to him when he needs it, other companies will respond to him. (5) The customer already coexists with the multi-channel, and he expects the same from the company, that his information, the state of his products and services, or the processes in progress, are visible and can be interacted with from any channel. (6) The company must change its focus, from business transactions to managing customers and connected social communities with which they can maximise their business transactions and opportunities.
- *Review of objectives* It is very important to define the general objectives that can benefit from the use of social software, as well as the strategy to be followed in order to achieve them, and the role that primary users (staff and key volunteers) should adopt (Dreyer and Grant 2010). An example of an objective is to increase customer loyalty by making use of social software.

### Customer strategy

Social CRM is focused on social customer management, as well as on identifying, attracting and achieving new social customers (Greenberg 2010). Social customers are those customers of the company that are managed using Social CRM. For them it is necessary to define the Social Customer Strategy, which is a part of the overall Customer Strategy.

Social customer strategy is defined by social customer engagement, not social customer management as in traditional CRM, which implies that there is a mutual benefit planned from the beginning (Mosadegh and Behboudi 2011). In order to define the Social CRM strategy, the company should take the following steps:

#### Identify social customers

First, the social customers must be identified from the current customers with whom collaboration and relationships of commitment by Social CRM can be set up. It is important to consider that building relationships with inappropriate customers is one of the main reasons for failure in projects of systems related to the customer (Hu et al. 2013; Lambert 2010). The identification of social customers can be accomplished by Big Data techniques of advanced analytics, since they help to discover trends, patterns and other insights, applied to historical information from past interactions with customers and public information on social networks.

#### Finding new social customers

One of the strengths of Social CRM is that it facilitates the search for new potential social customers that can contribute in the future to the enterprise in terms of branding, development or improvement of products or services, etc. In order to carry out this search it is very useful to perform social profile mapping, which is the process of collecting social data about people and their relationships, to know more about them and to analyse whether they or their contacts are potential possible customers for the company (Dreyer and Grant 2011a). Big Data tools of advanced analytics can be used to listen to and learn from the social media activity and apply the insights to identify possible customers.

#### Segmenting the social customers

The traditional methods of customer segmentation are based on characteristics such as age, gender, interests and consumer habits. However, Social CRM allows another form of segmentation, since it has a lot of information that can be gathered online and is growing quickly. To manage and analyse that vast amount of information in real time, with the aim of segmenting social customers, it is necessary to use Big Data tools (such as Apache Mahout for databases, and R as a programming language), with data mining techniques (Petz et al. 2014): clustering, classification, association, regression and visualisation. Regarding the types of segmentation, the most frequently used are: attitudinal, behavioural, demographic, loyalty and value-based (Fotaki et al. 2014).

Social customers can be organised in communities (social groups), which are groups of users linked by some kind of feature, relationship or common interest (Karrer et al. 2008; Wu et al. 2009). To do this, Social Network Analysis (SNA) tools are used, which provide mathematical and statistical routines that are applied to analyse the social networks, the results of which are represented in a social network diagram. The SNA employs community detection algorithms using the social contacts of individuals (Mosadegh and Behboudi 2011).

By being in the appropriate community, social customers can (a) attract new social customers, (b) retain other social customers, and (c) acquire a new product or service by being influenced by other social customers of the same community (McKay 2009; Serrat 2010). The segmentation process should be carried out as follows:

- First, organise social customer types into different segments based on the communities concept, using as criteria the enterprise products and/or services of interest to each social customer.
- Then, in the communities deemed necessary, other levels of segmentation can be considered to create sub-communities, using as criteria the characteristics that are thought to be appropriate, depending on each community.
- Finally, there is a last level of segmentation that identifies social customers who provide more value within the initial segment. Some possible criteria for segmentation at this level are: profitability, growth potential, volume, competitive positioning issues, access to market knowledge, market share goals, margin levels, level of technology, resources and capabilities, compatibility of strategies, channel of distribution, and buying behaviour (Lambert 2010).
- There must also be a periodic monitoring of the evolution of communities and sub-communities in order to better understand their life cycle and thus manage them more efficiently, and, if necessary, undertake the social customer segmentation once again (Karrer et al. 2008; Serrat 2010).

#### Analyse the profitability of social customers

The profitability and potential of each social customer and community that the company has is analysed. The profitability analysis is carried out not only in economic terms but also in relation to the image, productivity or any other benefit that the company can obtain as a result of the relationship with the social customers.

#### Define the objectives of social customers

The objectives that will be assigned to each social customer and each community, in the short, medium, and long term are defined. These objectives are established based on the profitability and potential of each social customer and community. Examples of social customer objectives might be to improve the image, collaboration in the design and development of products, generating revenue with more effective cross-marketing, cross-selling and/or up-selling, to reduce marketing costs, etc. In order to achieve this, the company must develop strategies that enable social and business customers to operate as a true community, thereby generating some feeling of belonging and loyalty from the social customers. Social interactivity with social customers must be enhanced to obtain new ideas and different points of view. This will allow the social customer to be known from another perspective, understanding their preferences and also their demands and needs in order to provide a better service and/or product. After defining the objectives for each social customer or community, the level of fulfilment of these objectives can be analysed through Big Data advanced analysis tools.

### System for assessing customer relations

The distinguishing characteristics of Social CRM compared to traditional CRM make it necessary to define new indicators that are not in the measurement system of a traditional CRM. These indicators should help to assess not only the degree of compliance with the needs and expectations of customers, as in traditional CRM, but also new aspects such as the degree of interaction, collaboration or commitment of social customers with the company, the involvement of company staff in Social CRM, or the business performance objective for Social CRM. The use of Big Data advanced analysis tools allows the defined indicators to be assessed both quickly and accurately. The indicator system of the Social CRM will be made up of:

- Traditional indicators like web page views, number of clicks, conversion rate and page or site “stickiness” (Greenberg 2010), although also taking into account aspects such as time spent on a page, the number of times a page has been visited by the same person and the number of page views per session.
- Generic social indicators such as (Greenberg 2010):
  - *Volume* The number of times a topic is mentioned compared to historical patterns.
  - *Tone* The percentages of positive, negative and neutral opinions.
  - *Coverage* The number of sources that are generating the conversation regarding a particular topic.
  - *Authorisation* Classification of sources by their level of authority, and observation of how many rises or falls of conversations are generated by authoritative sources.
- Specific social indicators to monitor aspects of Social CRM of interest, such as: the resolution time for queries, the number of posts, the accuracy of the answers, the number of participants, etc. (Sarner et al. 2010). And also the number of times an issue has been read on each channel, when and by which social customer, as well as whether it has been shared.
- Finally, the cause-effect relationships between the above indicators and the business objectives are defined.

### Process map

Initially, Social CRM was mostly a concern of marketing, but it now affects every customer-oriented discipline, from marketing and sales to customer service and support, as well as other internal company processes such as design, research, innovation, etc. Each of these enterprise business processes must be analysed, defining how they can benefit from Social CRM through Web 2.0 and Big Data technology. This will involve redesigning the processes, modifying or adding new activities within each process to consider the contributions of the social customer. These contributions can come from both direct feedback as well as in an indirect form by extracting knowledge about their emotions and behaviour from the profiles and maps of experience that are stored by the technological part of Social CRM (Mosadegh and Behboudi 2011). As an example, Table 2 shows the business processes of different departments which have been improved as a result of the implementation of the SCRM-IRIS methodology in the company of the case study.

**Table 2:** Business processes improved as a result of the implementation of the SCRM-IRIS methodology in the case study

Department	Business process
Strategic management	Real-time analysis of the competitive environment
	Detection of changes in the competitive environment
	Data-driven decision-making
	Strategic planning
Operations	Troubleshooting in the products/services offered
	Increasing the quality of the products/services offered
	Offering an efficient catalogue of products/services based on sales trends analysis
Research and development	Monitoring the performance and quality of the products/services offered
	Identifying the needs of customers of new products/services
	Identification of improvements in the products/services offered
Marketing/sales	Analysis of customer information
	Identification of potential customers
	Identification of the most valuable customers
	Analysis of competing companies
	Gathering information about customers’ needs
	Research about the company image
	Service acceptance analysis
	Monitoring social networks
	Price monitoring
	Detection of new releases by competing companies
	Analysis of relations in social networks
	Predicting customer behaviour
	Accurate prediction and awareness of customers’ needs
	Making real-time customised offers
	Encourage participation and interaction in every channel
	Quick reaction to market opportunities
	Analysis of sales trends
Customer assistance	Identifying customers who are at risk of ceasing to be customers of the company
	Analysis of how customers use the company website
	Monitoring how customers use the products/services offered by the company to detect potential problems and/or improvements

Moreover, due to the characteristics of Social CRM, there are several processes that did not exist in the company and had to be designed and implemented for the first time. These processes can be grouped into four areas (Dreyer and Grant 2011a): Social media monitoring and responding; Social mapping profile; Outreach and lead generation; and Community management.

### Human resources

Almost every department of the company must participate in the Social CRM. Each department will be responsible for the part that is related to their roles in the company. The communication department is generally the one that should monitor and assign the right people to respond in public social spaces (Dreyer and Grant 2011a).

Social software is not free, because time is money, and time must be invested in company staff working on a job that involves Social CRM. So it is important that much of this work is carried out by low-level staff, as their labour costs are lower. Lower-level staff can be trained with the necessary skills to enable them to manage social software applications, supported by the highest-level staff only when needed (Dreyer and Grant 2011b).

A social media policy must also be defined, which must be derived from the social media strategy adopted, and which aims to educate employees by providing guidance on how the company requires them to use the social software. This policy should focus less on the “don’ts” and more on the “do’s” and should facilitate and make the interaction with customers more pleasant and safer, as well as improving the ability to carry out the work. The main characteristics of good policies are: built on trust, practical, designed to educate, without absolutes, in plain language, friendly, consistent, prepared for mistakes, and clear about due process (Dreyer and Grant 2010).

One last thing to consider is the risk of employees using the social software for private use. While such use is made in the proper environment among employees and with partners and social customers, it can lead to a better business relationship. Controlling use is very difficult, so there must be trust and such control should not be undertaken (Assaad and Marx Gómez 2011).

### Computer system

This phase considers the Big Data and social software (or Web 2.0) applications and tools to be used in Social CRM. Both public (developed by other enterprises) and private (developed by the enterprise) ones should be taken into account, and those that will make up the computer system should be decided together with how they will be related to each other.

The computer support system of Social CRM is of great importance as it is the basis on which the Social CRM is run. This computer system has three basic objectives (Reinhold and Alt 2011): To create a tool to efficiently discover the opinions and user reviews about the company and its products or services; to establish a contact channel for two-way interaction with users of social software; and to provide the means to integrate social content from the social software to processes and systems oriented towards the social customer.

The minimum requirements and/or abilities that the computer system must have to achieve these objectives are: (a) Data storage, (b) Customer Profiles storage, (c) Social knowledge, advanced analysis and monitoring, (d) User Generated Content (Mosadegh and Behboudi 2011), and (e) User interaction (Reinhold and Alt 2011).

The computer system to support Social CRM will consist of a set of Big Data and social software applications, and a set of Big Data and social software tools.

The Big Data and social software applications are Wikis, Blogs, Social Networks, Hadoop, MapReduce, Cassandra, etc. The private Social CRM applications must be developed considering the following characteristics (Sarner et al. 2012):

- To make social customers feel more involved in their own decisions.
- To give social customers access to more and better information on products and services.
- To provide more control in managing the public image and reputation online as well as how to decide what personal information is to be used.
- To improve self-esteem, friendship, the level of respect and commitment of social customers.
- To encourage participation in many-to-many relationships with social customers, prospects, selling partners and employees.
- To capture and share user-generated data and content.
- To provide various levels of autonomy and commitment to cede control of the community.
- To demonstrate the existence of mutual and balanced benefits for both the company and the community.
- To understand the profile, needs and feelings of social customers about the products and/or services offered by the company.

Regarding the Big Data and social software tools, there are many types of tools that can be part of a Social CRM computer system. Below are the most important types of tools (Dreyer and Grant 2011a):

- *Social media monitoring* These filter the web content based on various characteristics, such as for example the mention of keywords or comments about a particular topic. The Big Data advanced analysis tools provide speed and accuracy in monitoring social media, allowing companies to have real-time information with which to make decisions.
- *Social media management/marketing systems* These manage the process of posting and responding through social media channels, facilitating and unifying such management across multiple channels.
- *Social discovery* These search for contacts in social media profiles and map the relationships between contacts, allowing information to be found from the contacts. In addition, they categorise, standardise and structure the big unstructured data existing in the social media, in order to harness the wisdom of crowds, without human intervention.
- *Email systems* These help to connect and communicate with contacts and customers by e-mail, creating and/or segmenting mailing lists using social discovery data and allowing information from social discovery data to be provided in e-mails.
- *Communities* These help to create social links through the website, offering various extra features to the contacts.
- *Association management systems* Databases where data such as contacts, interactions, transactions carried out, etc. are recorded.
- *Social network analysis* These analyse the social network links using graph theory and display the results in social network diagrams. This is a nice graphical way to analyse and visualise the large amount of existing data, as well as those generated every day. It is a part of Big Data that focuses on relationships and/or interactions between users of online social networks (Alamsyah and Peranginangin 2013).

**Fig. 2:**
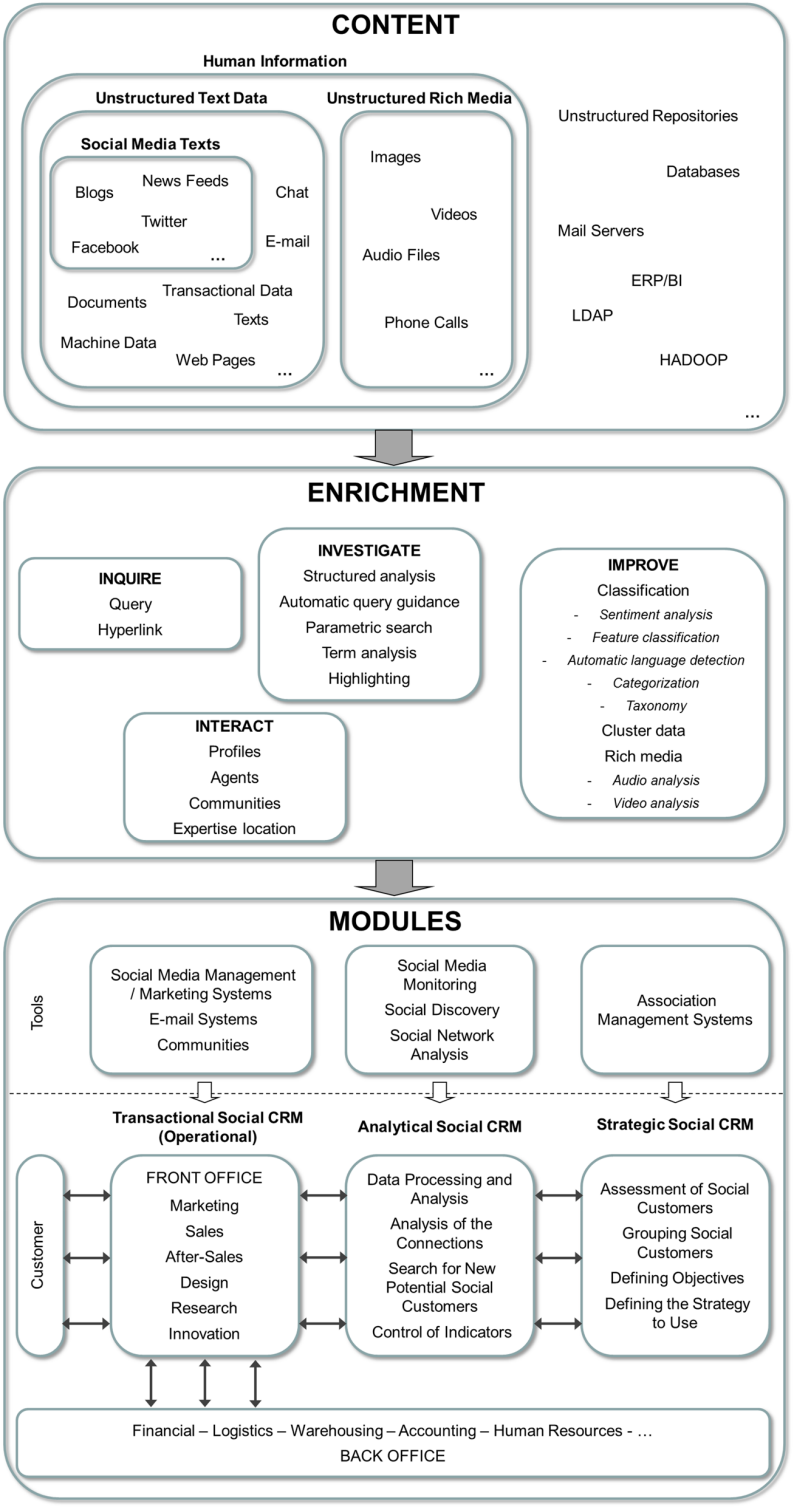
Social CRM computer system architecture

To support Social CRM, a Social CRM Computer System Architecture is proposed. It allows advantage to be taken of Web 2.0 and Big Data technologies (Fig. 2).

This Social CRM Computer System Architecture is composed of three levels:

- *Content* This level allows both structured and unstructured data to be gathered from different sources. At this level the links with the data sources are made by applying the necessary filters and patterns to obtain only valuable data for the company. Data sources can be both traditional computer systems (like corporate ERP, BI, structured databases, etc.) and social software that supports and stores human information and communication. The latter represents a major technological challenge because it makes it necessary to work with complex, unstructured, ubiquitous, multi-format and multi-channel information.
- *Enrichment* This level works with the data gathered by the Content level. The objective is to enhance the data and to identify and to extract valuable information for the company CRM from those data. For this purpose different advanced data analysis techniques are used. These techniques are organised in four groups: Inquire, Interact, Investigate and Improve. The result is valuable social data obtained from social software, links to valuable corporate data, and valuable new data (metadata) generated from the analysis of the previous data gathered at the Content level. These data are stored in a Social CRM structured database. Metadata are used to simplify and reduce the complexity and processing time at the next level (Modules level).
- *Modules* The enriched information extracted from the Enrichment level and stored in the Social CRM structured database is processed by the three main modules of the Social CRM software, using different techniques like data mining, predictive analysis or machine learning: (1) *Transactional Social CRM* (*Operational*). This supports the business processes that can be improved with the Social CRM: Marketing, Sales, After-sales, Design, Research, Innovation, etc.; (2) *Analytic Social CRM.* This allows data from social customers to be used to manage and improve relationships with existing social customers and achieve business objectives. Moreover, it also carries out an analysis of online social communities to find new potential social customers in these communities. Finally, it also allows measurement of the indicators of the Social CRM performance measurement system; and (3) *Strategic Social CRM*. This permits each social customer (profile, contacts, etc.) to be assessed and grouped in segments depending on their characteristics. In addition, it allows the definition of objectives for each segment in the short, medium, and long term, as well as definition of the strategy to be used in each segment to meet the proposed objectives.

### Implantation

The person in charge of the implantation should be the Community Manager, who should always be available to solve problems or queries arising from users, both in the implementation as well as when the system is operating. It is important that the response time in resolving problems or concerns is short.

Implementing a Social CRM system is very similar to the implementation of a CRM system; therefore, the steps to be followed in implementing a Social CRM system are the same as those shown in Chalmeta (2006). The collaboration of all Social CRM users in the implementation is critical, so it is essential that they change their mentality and assume that the centre of Social CRM is the social customer. On the other hand, in order to take advantage of Big Data advanced analysis tools, it is necessary to have good quality data. To ensure that the data are of good quality, organisations must maximise the following properties (Chiang and Sitaramachandran 2015): (a) *Existence*. The organisation has or can get the data; (b) *Validity*. The data values are within an acceptable range; (c) *Consistency*. The same data has the same value regardless of where it is located; (d) *Integrity*. Completeness relationships between data elements; (e) *Accuracy*. The data describe the properties of the model; and (f) *Relevance*. The data are appropriate to achieve the proposed objectives.

The person in charge of the creation and maintenance of the continuous improvement system is also the Community Manager, as he/she is the one who knows the entire system best and, therefore, is also better able to identify potential future improvements.

### Monitoring

Social CRM must be monitored throughout its entire lifespan. The Community Manager is in charge of carrying out this monitoring process. The characteristics of Web 2.0 and Big Data technologies allow the monitoring to be carried out quickly and effectively, while providing great control over the system.

In order to establish an effective monitoring system, the following tasks must be carried out:

- To monitor the indicators defined in stage four of the methodology so as to be able to carry out a follow-up of the system in order to measure its performance. To do so, the following technologies (which are based on Big Data advanced analysis tools) are used: Social Media Monitoring, Social Media Management/Marketing Systems and Association Management Systems.
- To adapt or modify the functionalities required, in order to fix errors and improve the system.
- To generate and maintain a system of periodic motivation for users, to reward users that make good use of the Social CRM system, since the participation of users is essential for Social CRM.
- To carry out a control of accesses and input from all users, as it is very important they make good use of the system.
- To carry out periodic user surveys to assess their level of satisfaction with the Social CRM, and to ascertain the level of acceptance of the system and compile their suggestions for improvement. After the results of the surveys have been obtained, they are studied and the relevant modifications are made.
- To periodically carry out an analysis of the system’s stability, which checks whether the Social CRM system is working properly, if it reacts correctly to the data it manages and if users use it properly.

## Conclusion

Organisations must be aware of the shift that is occurring in the use of data and must actively prepare to participate in it. Among the measures to be taken, three are absolutely critical: Treating information and data as a corporate asset at the same level as human and financial resources; The company should be capable of generating and sharing knowledge from the data; and Designing and implementing a technology infrastructure that makes it possible to address the challenges and opportunities presented by technological disruptors such as Security, Cloud, Mobility, and Big Data.

This paper presents a methodology, called the SCRM-IRIS methodology, to help companies to obtain value from data, by developing a Social CRM system. The methodology has been applied to a company in order to refine and validate it. Those responsible for the application of the SCRM-IRIS methodology in the company have indicated that the use of the SCRM-IRIS methodology has allowed them to gain an excellent view of the needs, scope, consequences and opportunities of the project, as well as allowing them to implement Social CRM quickly and without significant problems. They have also indicated that the methodology has enabled them to have greater control over the project, because all the activities to be performed in each phase of the implementation are clearly defined.

There are various proposals for future investigations. The future of Big Data and Web 2.0 technologies is going through a general expansion for all industries to be applied to all business processes and aspects of organisations. Through Big Data analytics and Web 2.0 technologies, the company can not only quickly and reliably monitor the acceptance of its products and/or services in the marketplace, but they also allow them to understand their business environment as well as find and strengthen competitive advantages (Kwon et al. 2014). Therefore, new methodologies, similar to the SCRM-IRIS methodology, are needed to support the adoption and implementation of Big Data and Web 2.0 technologies in other areas of the company, such as company strategy, supply chain management, product design, and so on.

Finally, some limitations of this paper should be noted and discussed. First, the qualitative method used for the analysis of the benefits obtained by the company from the application of the methodology is not as accurate as a quantitative analysis. The method is based on the opinion of those in charge at each implantation. However, their experience and professionalism make it possible to rely on the veracity of these results. Only a basic quantitative analysis was performed. This can be future research for academics that can apply advanced quantitative methods to measure the benefits of SCRM-IRIS at department level as well as business level. Finally, the company where the SCRM-IRIS methodology was applied already had CRM technologies (without Social CRM features). Therefore, if this methodology were to be applied in a company that did not have them, the implementation process would be more expensive and complex, because the implantation of CRM strategies, culture and computer systems, and training of the employees would have to be undertaken from scratch.
